# Natural Modulators of the PPARγ–Adiponectin Axis in Insulin Resistance: Structural Pharmacology, Receptor-State Selectivity, and Translational Readiness

**DOI:** 10.3390/ph19101507

**Published:** 2026-09-22

**Authors:** Pirscoveanu Denisa Floriana Vasilica, Diana-Maria Trasca, Adina Maria Kamal, Renata Maria Varut, Romeo Popa, Pluta Ion Dorin, Dirnu Rodica, Maria Stoica, Coanca Staicu Cristina Teodora, George-Alin Stoica

**Affiliations:** 1 Department of Neurology, Faculty of Medicine, University of Medicine and Pharmacy of Craiova, 200349 Craiova, Romania; denisa.pirscoveanu@umfcv.ro; 2 Department of Internal Medicine, University of Medicine and Pharmacy of Craiova, 200349 Craiova, Romania; diana.trasca@umfcv.ro; 3 Research Methodology Department, Faculty of Pharmacy, University of Medicine and Pharmacy of Craiova, 200349 Craiova, Romania; coancastaicucristinateodora@gmail.com; 4 Drug Research Center, University of Medicine and Pharmacy of Craiova, 200349 Craiova, Romania; 5 Department of Pharmacology, University of Medicine and Pharmacy of Craiova, 200349 Craiova, Romania; romeo.popa@umfcv.ro; 6 Faculty of Medical and Behavioral Sciences, Constantin Brâncuși University of Târgu Jiu, 210185 Târgu Jiu, Romania; dorin.pluta@e-ucb.ro (P.I.D.); rodica.dirnu@e-ucb.ro (D.R.); 7 Department of Intensive Care and Anesthesia, Emergency County Hospital, 200349 Craiova, Romania; maria.stoica@umfcv.ro; 8 Department of Pediatric Surgery, Faculty of Medicine, University of Medicine and Pharmacy of Craiova, 200349 Craiova, Romania; alin.stoica@umfcv.ro

**Keywords:** PPARγ, adiponectin, insulin resistance, selective PPARγ modulator, natural products, receptor phosphorylation, receptor acetylation, AdipoR1, AdipoR2, translational pharmacology

## Abstract

Insulin resistance reflects maladaptive communication among adipose tissue, the liver, skeletal muscle, immune cells, and the vasculature. Peroxisome proliferator-activated receptor gamma (PPARγ) is a validated insulin-sensitizing target, but classical full agonism couples metabolic efficacy to weight gain, fluid retention, and skeletal liability. This critical narrative review evaluates plant-derived and dietary molecules through a receptor-state framework that distinguishes direct binding from reporter activation and separates full agonism, partial agonism, selective modulation, antagonism with retained metabolic function, and post-translational control of PPARγ. Adiponectin is considered a functional inter-organ circuit linking adipocyte PPARγ activity to AdipoR1/AdipoR2 signaling, AMPK, PPARα, ceramide metabolism, glucose disposal, and fatty-acid oxidation. Amorfrutins provide the most coherent target-led natural-product evidence, whereas chelerythrine offers a structurally defined selective-modulation benchmark; honokiol, diosmin, betulinic acid, valerenic acid, punicic acid, alkamides, and defined extracts show heterogeneous mechanistic and translational maturity. Human evidence linking natural PPARγ ligands to verified target engagement and improved insulin sensitivity remains limited. We therefore propose a seven-stage translational roadmap connecting chemical identity, orthogonal binding, receptor-state pharmacology, cellular function, tissue exposure, mechanism-matched safety, and randomized human proof.

## 1. Introduction

Insulin resistance is not a single molecular defect. It is a systems-level failure involving adipocyte lipid storage, adipokine secretion, hepatic glucose production, skeletal-muscle glucose uptake, immune signaling, and mitochondrial substrate handling. Among the transcriptional regulators positioned at this interface, peroxisome proliferator-activated receptor gamma (PPARγ) is unusually well validated: thiazolidinedione ligands improve insulin sensitivity, yet their clinical utility is constrained by weight gain, edema, heart-failure risk in susceptible patients, and adverse skeletal effects. The central medicinal-chemistry problem is therefore not whether PPARγ can be activated, but whether its insulin-sensitizing program can be separated from the transcriptional programs that produce unwanted tissue effects.

The apparent paradox of an adipogenic receptor producing systemic insulin sensitization is resolved partly by adipose-tissue expandability. When adipocytes retain the capacity to esterify fatty acids, remodel extracellular matrix, and generate insulin-sensitizing adipokines, they can protect liver and skeletal muscle from lipotoxic intermediates. Conversely, dysfunctional adipose expansion promotes ectopic lipid, inflammation, and impaired insulin action. PPARγ therefore controls both a potential liability—fat storage—and a protective buffering program; the therapeutic objective is therefore to preferentially stabilize receptor states that preserve insulin sensitization while limiting adverse transcriptional programs [1,2,3].

Natural products offer a structurally diverse set of ligands for this question. Their value does not arise from being natural per se. Rather, many occupy the large PPARγ ligand-binding pocket differently from classical full agonists and may produce partial agonism, selective cofactor recruitment, antagonism, or modulation of receptor phosphorylation. The crystal structure of the human PPARγ ligand-binding domain revealed a large, accessible pocket and an extended Ω-loop, providing a structural basis for multiple binding modes [4]. Later work showed that obesity-linked phosphorylation of PPARγ at Ser273 can dysregulate an insulin-sensitizing gene set without being identical to classical receptor activation [5,6]. These findings changed the target concept from maximal transcriptional agonism to selective correction of receptor state.

Adiponectin is a particularly useful functional bridge between receptor pharmacology and whole-body metabolism. PPARγ ligands increase adiponectin expression and circulating concentrations [7], while adiponectin receptors activate pathways associated with glucose uptake and fatty-acid oxidation [8]. A compound that binds PPARγ but fails to improve adiponectin signaling, glucose disposal, or lipid partitioning is therefore mechanistically incomplete. Conversely, an increase in adiponectin without verified receptor engagement cannot establish that PPARγ is the causal target. This review uses the PPARγ–adiponectin axis as a disciplined framework for distinguishing target-based leads from broad phytochemical claims.

The focus is deliberately narrower than a general review of antidiabetic plants. It addresses molecular interaction with PPARγ, the relationship between receptor state and adiponectin output, evidence for glucose uptake and insulin sensitization, and the experimental steps needed to translate a plant-derived ligand into a reproducible pharmaceutical or nutraceutical candidate. Botanical reports based only on glycemia in chemically induced diabetes, antioxidant capacity, docking, or PPARγ mRNA abundance are considered supportive at most, because none of these observations independently demonstrates receptor-mediated insulin sensitization.

Several broad surveys have catalogued natural PPAR ligands, and a recent review focused specifically on ligands that inhibit PPARγ Ser273 phosphorylation [9,10]. The present review is differentiated by integrating three layers that are often treated separately: structural and post-translational receptor pharmacology; adiponectin/AdipoR signaling as a functional output; and a product-level translation framework that requires the same chemically defined material to connect molecular assays, tissue exposure, safety, and human metabolic outcomes.

## 2. Literature Approach and Evidence-Assessment Framework

A targeted narrative search of PubMed was conducted and updated on 28 August 2026. Search concepts combined PPARγ, selective PPARγ modulators, partial agonists, receptor-state modulation, Ser273 phosphorylation, adiponectin, AdipoR1, AdipoR2, AMPK, insulin resistance, glucose uptake, phytochemical, plant extracts, flavonoids, terpenoids, alkaloids, and natural products. Reference lists of relevant primary studies and reviews were additionally screened to identify mechanistically informative studies. Priority was given to primary studies that evaluated direct ligand binding, receptor structure, cofactor recruitment, transcriptional activity, receptor phosphorylation or acetylation, adiponectin secretion, GLUT4-dependent glucose uptake, adipogenesis, in-vivo insulin sensitivity, pharmacokinetics, or randomized human outcomes. Reviews were used to map the field and identify emerging concepts, whereas mechanistic conclusions were anchored whenever possible to primary experimental studies.

The search was designed for a critical narrative review rather than a systematic effectiveness review or meta-analysis. Eligible evidence included purified-receptor and structural studies, receptor-dependent cellular experiments, animal studies incorporating metabolic or safety phenotyping, human pharmacology, and randomized clinical studies. Candidate-level discussion was restricted to natural compounds for which the evidence extended beyond molecular docking or changes in PPARγ expression and included at least one experimentally supported target-level feature, such as direct ligand binding, structural evidence, receptor-state pharmacology, or receptor-dependent functional activity. Reports based only on molecular docking, antioxidant activity, PPARγ mRNA or protein abundance, or glycemic changes in chemically induced diabetes were not considered sufficient to establish direct PPARγ ligand activity. Representative candidates were prioritized when the same chemically defined material connected two or more evidence levels within the framework described below. Accordingly, the compounds discussed in detail are intended as mechanistic benchmarks rather than as an exhaustive catalogue of natural products reported to influence PPARγ. This selection strategy was chosen to emphasize continuity between chemical identity, receptor engagement, functional phenotype, exposure, and translational readiness. Operationally, a docking-only study was defined as one in which PPARγ engagement was inferred exclusively from an in-silico pose, docking score, or predicted interaction without orthogonal binding or structural evidence, receptor-dependent cellular validation, or a mechanism-matched functional assay; such studies were excluded from candidate-level synthesis.

Evidence was interpreted across five linked levels: chemical identity; direct target engagement; cellular function; whole-animal metabolic efficacy and safety; and human exposure or clinical response. This ordering matters. A molecular-docking score does not establish binding, reporter activation does not establish selective modulation, increased PPARγ expression does not establish ligand activity, and improved glucose tolerance does not prove that PPARγ caused the effect. Strong candidates connect several levels using the same chemically defined material.

Evidence strength was judged by convergence, not by the apparent magnitude of a single effect. Direct binding requires an orthogonal biophysical or structural method; receptor dependence requires pharmacological blockade, knockdown, mutation, or rescue; selective modulation requires comparison of cofactor recruitment, transcriptional programs, phosphorylation, acetylation, and adipogenesis; and translational plausibility requires measured unbound exposure at the relevant tissue. Human efficacy without product identity or target-relevant biomarkers was classified as axis-associated rather than target-confirmed. The evidence hierarchy is summarized in Table 1.

## 3. PPARγ as a State-Dependent Metabolic Target

PPARγ is a ligand-activated nuclear receptor that heterodimerizes with retinoid X receptor and regulates genes through peroxisome proliferator response elements. Its physiological role extends beyond adipocyte differentiation. It controls fatty-acid uptake and esterification, adipokine production, inflammatory tone, extracellular-matrix remodeling, and the capacity of adipose tissue to buffer nutrient excess. This buffering can protect liver and muscle from ectopic lipid, even when adipose mass increases. Genetic studies illustrate the resulting paradox: reducing adipose PPARγ may limit fat expansion, yet severe or context-specific loss can produce lipodystrophy, hepatic lipid accumulation, and insulin resistance. Adipose-specific knockout models therefore caution against a simple model in which less PPARγ is always metabolically favorable [14].

### 3.1. Domain Architecture, Canonical Binding, and the Alternate Site

PPARγ contains an N-terminal activation domain, a DNA-binding domain, a flexible hinge, and a C-terminal ligand-binding domain. The ligand-binding domain is unusually large and branched, allowing chemically diverse ligands to occupy different subpockets. Classical thiazolidinediones stabilize helix 12 and the activation-function-2 surface, favoring strong coactivator binding. The structural consequence is not merely receptor occupancy but redistribution of a conformational ensemble, which explains why ligands with comparable affinity can produce different transcriptional outputs [1,4].

An alternate solvent-accessible site adjacent to helix 3 and the Ω-loop can accommodate a second ligand or a ligand pose that does not reproduce canonical helix-12 stabilization. Nuclear magnetic resonance, mutagenesis, and crystallography have shown that alternate-site occupancy can affect coregulator binding, transactivation, and receptor phosphorylation at pharmacologically relevant concentrations [15,16]. This architecture supports allosteric fine-tuning but also creates an assay problem: a single reporter curve cannot reveal site occupancy, stoichiometry, or conformational heterogeneity.

### 3.2. Full Agonism, Partial Agonism, and Functional Selectivity

A full agonist stabilizes the canonical activation surface and strongly recruits coactivators, but receptor occupancy and transcriptional amplitude do not map linearly onto insulin sensitization. Selective PPARγ modulators can generate a different cofactor profile, alter only a subset of target genes, or improve glucose uptake with little adipogenesis. An early synthetic selective modulator blocked adipocyte differentiation while stimulating glucose uptake in 3T3-L1 adipocytes, demonstrating that these outcomes can be pharmacologically separated [17]. Natural products are attractive in this context because their scaffolds frequently contact the receptor outside the interaction pattern typical of thiazolidinediones.

Partial agonism is nevertheless not a synonym for safety. A ligand may show weak reporter activation but still promote adipogenesis, interact with PPARα or RXR, or reach insufficient tissue concentrations. Conversely, antagonism in a reporter assay does not exclude beneficial receptor-dependent actions. Betulinic acid, for example, bound PPARγ and PPARα as an antagonist, inhibited adipogenesis, promoted osteogenesis, and increased basal glucose uptake in adipocytes [18]. The result is important conceptually, but cellular glucose uptake does not yet establish durable systemic insulin sensitization or clinical safety.

Here, receptor-state selectivity refers to differential stabilization of PPARγ conformational, coregulator, and post-translational states rather than to biased agonism in the formal GPCR sense. Coregulator recruitment, chromatin occupancy, adipocyte gene expression, lipid accumulation, adiponectin release, glucose transport, and anti-inflammatory transrepression may diverge among ligands. A candidate should therefore be compared with a full agonist and an antagonist across matched receptor occupancy or unbound concentration. Otherwise, low efficacy may reflect poor exposure, assay interference, or receptor-independent signaling rather than genuine receptor-state selectivity.

Recent structure–function studies further reinforce the concept that PPARγ activity reflects a conformational ensemble rather than a simple occupied-versus-unoccupied receptor state. Non-covalent inverse agonists have been shown to preserve or improve insulin-sensitizing activity while producing receptor conformations distinct from classical agonism, and recent structural analyses have identified determinants governing this inverse-agonist behavior. These findings provide contemporary synthetic benchmarks against which claims of natural-product receptor-state selectivity should be evaluated [19]. The operational distinction between these pharmacological frameworks is summarized in Table 2.

### 3.3. Post-Translational Control: Phosphorylation, Acetylation, and SUMOylation

Obesity activates cyclin-dependent kinase 5 and related kinase pathways that phosphorylate PPARγ at Ser273. This modification changes a metabolic gene program, including reduced adiponectin expression, without simply switching off adipogenesis [5]. Non-agonist ligands can block the phosphorylation event and improve insulin sensitivity in mice, providing proof that classical agonism is not required for an antidiabetic effect [20]. Genetic prevention of PPARγ phosphorylation also preserved insulin sensitivity in diet-induced obesity and was associated with smaller adipocytes, higher adiponectin, and lower circulating free fatty acids [21].

The pathway has since been refined. PPARγ Ser273 phosphorylation promotes insulin resistance in part through growth differentiation factor 3, as shown by non-phosphorylatable receptor genetics, transcriptomics, and metabolic clamp experiments [6]. A more recent non-agonist ligand, AM-879, improved insulin sensitivity at an effective dose while blocking Ser273 phosphorylation in mice, although dose-dependent effects and the absence of human evidence remain important constraints [22]. Structural studies of alternate-site binding near the Ω-loop further show how a ligand can influence phosphorylation and transactivation differently [16]. These observations provide a rigorous assay set for natural-product research: direct binding, Ser273 status, gene-selective transcription, adiponectin output, and glucose disposal should be measured together.

Ser273 is Ser245 in PPARγ1 numbering, a distinction that should be stated explicitly when comparing structural and cellular studies. Protein phosphatase PPM1A can directly dephosphorylate Ser273 and restore a substantial portion of the dysregulated gene program, while phosphomimetic experiments indicate that phosphorylation changes the dynamics of coactivator and corepressor recruitment [23,24]. These findings make phosphorylation a receptor-state biomarker, but not a self-sufficient surrogate for whole-body benefit. Natural-product-inspired medicinal chemistry provides a complementary benchmark. A chrysin-derived selective PPARγ modulator, YGT-31, was reported to inhibit Ser273 phosphorylation while increasing adiponectin and GLUT4-related signaling and improving glucose and hepatic phenotypes in preclinical models. Because YGT-31 is a chemically optimized derivative rather than an unmodified natural constituent, it is best considered a proof of concept for scaffold optimization rather than a natural-product candidate within the evidence hierarchy used here [25].

Acetylation at Lys268 and Lys293 provides an additional selectivity axis. SIRT1-dependent deacetylation recruits PRDM16, supports browning of white adipose tissue, and can separate insulin-sensitizing effects from classical thiazolidinedione liabilities in genetic models [26,27]. More recent work links the acetylation state to circadian adipose plasticity, brown-adipose whitening, aging, and adverse skeletal responses [28,29]. Natural compounds that influence SIRT1 or cellular acetyl-CoA cannot, however, be described as direct PPARγ deacetylation modulators unless receptor-site measurements and causality experiments are provided.

PPARγ also participates in SUMOylation-dependent transrepression of inflammatory genes. Ligand-dependent stabilization of a corepressor complex can suppress selected inflammatory promoters without mirroring canonical adipogenic activation [30]. For natural-product programs, anti-inflammatory activity should therefore be mapped to receptor dependence and cell type; generalized suppression of NF-κB or oxidative stress is not evidence of PPARγ transrepression.

### 3.4. Tissue- and Cell-Type Context

Adipocytes are the principal metabolic context for the axis, but PPARγ in macrophages, stromal cells, the endothelium, bone, the kidney, and the collecting duct contributes to both efficacy and liability. Macrophage PPARγ supports alternative activation and can improve insulin resistance in obese mice [31]. In the kidney, receptor activation contributes to sodium handling and fluid retention; in bone marrow, altered lineage allocation can favor adipocytes over osteoblasts. A compound described as selective in one adipocyte line may therefore be non-selective at the organism level.

Adipose expansion itself is not a binary adverse endpoint. Hyperplastic expansion with smaller insulin-responsive adipocytes can be metabolically safer than hypertrophic, inflamed expansion with lipid spillover. Development studies should distinguish adipocyte number, size, depot, extracellular-matrix remodeling, lipolysis, and ectopic lipid rather than using total fat mass as the only readout [2,3]. The main receptor-state concepts are summarized in Figure 1.

## 4. Adiponectin as an Inter-Organ Functional Circuit

### 4.1. Secretion, Multimerization, and Tissue Presentation

Adiponectin is secreted predominantly by adipocytes and circulates in several oligomeric forms. Its concentrations generally decline with visceral obesity and insulin resistance, despite expansion of adipose mass. PPARγ activation can increase adiponectin transcription and secretion in cultured adipocytes, rodents, and insulin-resistant humans [7]. This response is valuable because it links an adipose nuclear receptor to metabolic effects in liver and muscle, but total adiponectin concentration is an incomplete biomarker. High-molecular-weight adiponectin, receptor expression, tissue responsiveness, and the metabolic context influence the downstream signal.

Adiponectin circulates as low-, middle-, and high-molecular-weight assemblies, and the distribution among oligomers can change independently of total concentration. High-molecular-weight adiponectin is often more closely associated with insulin sensitivity, but assay harmonization, sex, renal function, adiposity, and medication influence interpretation. T-cadherin can retain hexameric and high-molecular-weight adiponectin on cardiovascular tissues, adding a tissue-presentation layer that is not captured by plasma concentration alone.

### 4.2. AdipoR1/AdipoR2 Signaling, AMPK, PPARα, and Ceramide

AdipoR1 and AdipoR2 are structurally distinct seven-transmembrane receptors. Their cloning established that they mediate adiponectin-dependent activation of AMPK and PPARα-associated pathways, fatty-acid oxidation, and glucose uptake [8]. Crystal structures revealed an internal zinc-coordinating cavity and an orientation distinct from G-protein-coupled receptors [32]. The adaptor protein APPL1 connects adiponectin receptors to lipid oxidation, insulin signaling, and GLUT4 translocation [33]. In primary adipocytes, globular adiponectin increased glucose uptake through AMPK even without canonical insulin-receptor or Akt activation [34]. Together, these studies explain why adiponectin can extend a PPARγ signal from adipose tissue to systemic substrate handling.

Adiponectin receptor signaling extends beyond AMPK. AdipoR1 and AdipoR2 possess or support ceramidase activity that lowers ceramide and generates sphingosine, which can be converted to sphingosine-1-phosphate. Genetic and biochemical evidence connects this lipid-remodeling activity to improved insulin action and cell survival [35,36]. The pathway is mechanistically important because ceramide can inhibit Akt signaling and link nutrient excess to insulin resistance.

The synthetic agonist AdipoRon provides a receptor-dependence benchmark: it bound AdipoR1/2, activated AMPK and PPARα pathways, improved glucose intolerance in obese mice, and lost its metabolic effect in AdipoR1/AdipoR2 double-knockout animals [37]. Natural-product studies claiming direct AdipoR agonism should be held to a comparable standard rather than inferring receptor activation from AMPK phosphorylation alone.

### 4.3. From Adipocyte Output to Systemic Insulin Sensitivity

The axis should not be reduced to a single linear arrow. PPARγ regulates many adipocyte genes in addition to adiponectin; adiponectin signals through more than one receptor and may act differently across muscle, liver, adipose tissue, vasculature, and heart. Hyperglycemia and hyperinsulinemia can also alter receptor abundance and adiponectin responsiveness. A candidate that increases adiponectin in 3T3-L1 cells may therefore fail if it is not absorbed, if it produces dysfunctional adipose expansion, or if target tissues are resistant to adiponectin. Conversely, gut-restricted or microbiome-mediated interventions can increase circulating adiponectin without directly binding PPARγ.

A receptor-agonist antibody provides an instructive non-botanical benchmark: it activated AMPK, glucose uptake, and fatty-acid oxidation in muscle cells [38]. The study confirms that the downstream receptor arm is independently druggable. For natural-product development, this means that increases in adiponectin secretion should ideally be paired with functional assays in AdipoR-expressing myotubes or hepatocytes and with antagonist, knockdown, or rescue experiments that distinguish PPARγ-dependent secretion from direct AdipoR agonism.

A convincing axis experiment therefore has three linked components: receptor-state evidence in adipocytes; secretion and oligomerization of adiponectin; and receptor-dependent metabolic function in a target tissue. Conditioned-medium transfer, adiponectin neutralization, AdipoR knockdown, and rescue with recombinant adiponectin can distinguish a true endocrine circuit from parallel direct effects of the candidate on muscle or the liver. Importantly, the PPARγ–adiponectin relationship should not be interpreted as obligatory or strictly linear. PPARγ can influence insulin sensitivity through adiponectin-independent programs, whereas circulating adiponectin can be modified by interventions that do not directly engage PPARγ. Accordingly, adiponectin should be treated as a mechanistically informative component of the axis rather than as a surrogate for PPARγ target engagement. The integrated circuit is illustrated in Figure 2.

## 5. Natural-Product Ligand Classes and Representative Candidates

The natural-product literature contains flavonoids, stilbenoids, neolignans, terpenoids, fatty acids, alkamides, and complex plant extracts reported to modulate PPARγ. A field-level survey concluded that many identified natural ligands behave as partial or selective modulators and may also engage PPARα, PPARβ/δ, or RXR [9]. Polypharmacology may be beneficial, but it complicates causal attribution. The most informative candidates are those with a defined chemical structure, orthogonal binding evidence, a receptor-pharmacology profile, and metabolic phenotypes measured at concentrations consistent with exposure.

### 5.1. Amorfrutins: The Strongest Target-Led Natural Scaffold

Amorfrutins isolated from edible leguminous plants are among the strongest examples of target-led natural-product discovery. Amorfrutin 1 bound PPARγ, produced a selective transcriptional profile in primary human adipocytes, and improved insulin resistance and inflammatory-metabolic parameters in diet-induced obese and db/db mice without the fat accumulation typical of full agonists [39]. Amorfrutin B showed low-nanomolar PPARγ binding, weaker interactions with other PPAR isoforms, selective cofactor behavior, and glucose-lowering activity in insulin-resistant mice without detected weight gain, fluid retention, or adverse osteoblast effects in the tested systems [40].

These studies set a useful standard but do not complete translation. The in-vivo doses were high relative to ordinary dietary exposure, and human pharmacokinetics, formulation performance, metabolite activity, and long-term safety remain insufficiently characterized. Amorfrutins are best viewed as validated scaffolds and mechanistic benchmarks rather than established nutritional treatments. Their main contribution is proof that a plant-derived ligand can generate a receptor program distinguishable from thiazolidinedione full agonism.

Their development value lies in the coherence of the evidence chain: isolated chemistry, direct receptor binding, selective transcriptional behavior, primary human adipocyte data, and efficacy in more than one insulin-resistant mouse model. What remains missing is equally informative—absolute oral bioavailability, unbound adipose exposure, active-metabolite profiling, chronic cardiovascular and skeletal safety, and randomized human proof. Amorfrutins therefore define the preclinical benchmark against which less characterized botanical candidates should be compared.

### 5.2. Honokiol, Valerenic Acid, and Punicic Acid

Honokiol, a neolignan associated with Magnolia bark, directly bound the PPARγ ligand-binding domain and acted as a partial agonist. It stimulated basal glucose uptake to an extent comparable with pioglitazone in adipocytes but did not induce the same adipogenic differentiation in the tested cell models [41]. The result supports a non-adipogenic ligand profile, although the contribution of RXR and other targets, systemic exposure, and chronic toxicology require careful separation.

Honokiol also improved hyperglycemia and limited weight gain in diabetic mice in the original target-led program, but its pleiotropic pharmacology complicates attribution [41]. Independent reports have described effects on insulin signaling, macrophage polarization, hepatic inflammation, and mitochondrial pathways. Such pleiotropy may be useful clinically, yet only exposure-matched receptor-loss experiments can determine how much of the in-vivo phenotype is mediated by PPARγ rather than parallel targets.

Valerenic acid, a marker compound of Valeriana officinalis, activated PPARγ weakly, increased adiponectin and GLUT4 expression, promoted adipocyte differentiation, and increased glucose uptake in 3T3-L1 adipocytes [13]. Its probable binding near the Ω-loop is mechanistically interesting because alternate-site engagement can generate partial agonism. At the same time, the study illustrates why partial agonism cannot automatically be described as non-adipogenic: valerenic acid promoted differentiation in the experimental model. A development decision would require comparing the magnitude of glucose uptake, adiponectin secretion, lipid accumulation, and receptor phosphorylation across matched concentrations.

Punicic acid, a conjugated fatty acid abundant in pomegranate seed oil, moderately promoted adipogenic markers but also increased GLUT4 expression, translocation, glucose uptake, and adiponectin secretion in adipocytes [42]. Its lipid nature creates a different translational problem from that of a rigid small-molecule scaffold: free concentration, incorporation into cellular lipids, oxidation products, and dietary matrix may dominate apparent potency. The relevant comparison is therefore not only with rosiglitazone in a reporter assay, but also with achievable unbound tissue exposure after a defined formulation.

### 5.3. Chelerythrine: A Structurally Defined Selective PPARγ Modulator

Chelerythrine provides one of the clearest structural examples of a natural-product scaffold producing selective PPARγ pharmacology. Competitive binding experiments demonstrated direct PPARγ engagement, with an IC_50_ of approximately 566 nM, while structural analysis revealed a binding mode oriented parallel to helix 3 rather than the classical thiazolidinedione interaction pattern. The PPARγ–chelerythrine complex has been structurally characterized (PDB ID: 4Y29). Chelerythrine destabilized helix 12, particularly the Tyr473-associated activation surface, produced a cofactor-recruitment profile distinct from classical full agonists, and showed substantially weaker classical transcriptional agonism despite direct receptor binding [43].

Functionally, chelerythrine inhibited CDK5-mediated PPARγ phosphorylation and improved insulin sensitivity in db/db mice, providing a direct connection between structural receptor engagement, selective receptor-state regulation, and an in-vivo metabolic phenotype. This combination makes chelerythrine particularly relevant to the receptor-state framework proposed here. Nevertheless, the evidence remains preclinical, and systemic pharmacokinetics, long-term safety, clinically relevant tissue exposure, and randomized human evidence remain insufficient. Chelerythrine should therefore be regarded as a structurally validated mechanistic benchmark rather than as an established therapeutic natural product.

### 5.4. Diosmin and Betulinic Acid: Selectivity Beyond Partial Agonism

Diosmin provides a recent example of selective modulation. Biophysical and molecular experiments indicated direct PPARγ binding, weak transcriptional activity, and inhibition of receptor phosphorylation. Local administration to inguinal white adipose tissue in mice improved insulin sensitivity, promoted browning, reduced inflammation, and attenuated hepatic steatosis [44]. The local route was valuable for proof of mechanism but bypassed oral absorption and distribution; it therefore cannot be treated as evidence that an oral diosmin product will reproduce adipose target engagement.

Betulinic acid illustrates the related possibility of antagonism with retained metabolic function. Structural and cell-based work identified an unusual binding mode and showed increased basal glucose uptake, reduced adipogenesis, and increased osteogenesis [18]. These effects are attractive in relation to weight and bone concerns, but receptor antagonism may produce tissue- and context-dependent outcomes. The next decisive experiments are oral pharmacokinetics, target engagement in adipose tissue, insulin-clamp phenotyping, and chronic comparison with a full agonist under exposure-matched conditions.

Diosmin and betulinic acid are conceptually important because neither fits a simple continuum from weak to strong agonism. Diosmin combines direct binding, weak transactivation, and phosphorylation blockade, whereas betulinic acid combines antagonistic receptor behavior with glucose uptake and anti-adipogenic effects. Their translational challenges differ: diosmin must overcome the gap between local adipose delivery and oral exposure, while betulinic acid requires demonstration that antagonism does not compromise adaptive adipose buffering in chronic metabolic stress.

### 5.5. Isolated Constituents, Extracts, and Chemically Defined Fractions

These extract-level examples are included to illustrate the breadth of discovery-stage evidence and hypothesis generation rather than to imply that they meet the evidentiary threshold applied to the more developed, chemically defined candidates.

Screening approaches have identified plant extracts that activate PPAR isoforms and increase glucose uptake with limited adipogenesis. A survey of 263 species found extracts of Terminalia chebula and Thymelaea hirsuta with dual PPARα/γ activity, increased cellular glucose uptake, and little adipocyte differentiation under the tested conditions [11]. An earlier multi-assay platform similarly identified common food and medicinal plants that activated PPARγ and enhanced insulin-stimulated glucose uptake with variable effects on differentiation [12]. Such screens are useful for prioritization because they compare metabolic function with adipogenic liability, but active constituents, assay interference, and batch variability remain unresolved until bioassay-guided isolation is completed.

Extract-level studies also illustrate the transition from broad phenotypic signals to pathway-directed hypotheses. Boehmeria nivea was reported to increase glucose uptake in C2C12 cells through PPARγ-associated signaling and to improve glucose intolerance in high-fat-fed mice [45]. Chamomile flower extract stimulated PPAR-family nutrient-sensor transcription in obese mice [46], while curcumin was linked to interacting PPARγ, NF-κB, and AMPK pathways in diabetic mouse liver [47]. Punica granatum flower extract was reported to activate PPARγ and yielded an active constituent during fractionation [48]. These studies broaden chemical and botanical coverage, but extract composition, direct receptor binding, and exposure-matched controls are uneven; they should be treated as mechanistic leads rather than equivalent demonstrations of selective PPARγ modulation.

Three seed-derived compounds from Swietenia macrophylla displayed PPARγ ligand activity, induced adipocyte-related genes including adiponectin, and promoted GLUT4-linked glucose uptake while producing fewer lipid droplets than rosiglitazone [49]. Isomeric C12 alkamides from Echinacea purpurea also behaved as partial PPARγ agonists and increased basal and insulin-dependent glucose uptake in adipocytes [50]. These examples reinforce the importance of testing isolated constituents rather than assigning an extract-level phenotype to a familiar marker compound. They also show why adiponectin, GLUT4, and lipid accumulation should be interpreted together rather than reported as independent positive endpoints.

Extract studies are most valuable when they function as discovery pipelines rather than final therapeutic claims. Bioassay-guided fractionation should retain quantitative mass balance, while orthogonal analytical methods should identify active constituents and exclude pan-assay interference. The same quantitative fingerprint must then be carried into pharmacokinetic, toxicological, and efficacy experiments; otherwise, an extract name creates continuity that the chemistry does not support. The representative candidate evidence is summarized in Table 3.

## 6. Human Evidence and Clinical Benchmarks

### 6.1. Natural Candidates: Axis Association Is Not Target Confirmation

Human evidence directly connecting a plant-derived PPARγ ligand to receptor engagement, adiponectin induction, and improved insulin sensitivity is sparse. An earlier study associated the hypoglycemic effects of jujube, barberry, and saffron with serum adiponectin, but an association across multi-constituent foods cannot identify PPARγ as the causal target [51]. A randomized crossover trial of phytate supplementation in 39 people with type 2 diabetes reported increased adiponectin and lower HbA1c after three months [52]. The result is clinically relevant as proof that a dietary compound can alter adiponectin alongside glycemic control, but it does not establish direct PPARγ binding, and several gut, mineral, inflammatory, or dietary mechanisms could contribute. It should therefore be classified as axis-associated human evidence rather than target-confirmed PPARγ pharmacology.

For translation, circulating adiponectin should be paired with a prespecified metabolic endpoint and an exposure measure. High-molecular-weight adiponectin may be more informative than total concentration, while insulin sensitivity should ideally be quantified by clamp, frequently sampled tolerance testing, or a validated surrogate interpreted with its limitations. Adipose-tissue biopsies, when feasible, can assess PPARγ target genes, receptor phosphorylation, and inflammatory remodeling. Without such triangulation, a rise in adiponectin can function as a pharmacodynamic clue but not as proof of the proposed mechanism.

At present, no natural candidate reviewed here has a complete human chain linking verified systemic exposure, adipose PPARγ target engagement, a receptor-state biomarker, adiponectin/AdipoR function, and improved insulin sensitivity. This absence should be stated explicitly. It does not invalidate the preclinical leads; it defines the next development step and prevents a review from converting mechanistic plausibility into clinical recommendation.

### 6.2. Synthetic Selective Modulators as Human Proof of Principle

Human experience with synthetic selective modulators demonstrates that receptor-state selectivity can be clinically meaningful but dose dependent. In a 24-week randomized trial of 367 participants with type 2 diabetes, INT131 produced dose-related HbA1c reductions; at a lower effective dose it caused less edema, weight gain, and hemodilution than high-dose pioglitazone, whereas higher doses narrowed the safety advantage [53]. This result warns against labeling a molecule intrinsically safe without defining the exposure window.

Balaglitazone, a partial agonist, also lowered HbA1c in a 26-week phase III comparison involving 409 insulin-treated participants, with a differentiated but not liability-free profile [54]. These trials provide two benchmarks for natural ligands: selective pharmacology must survive oral dosing and chronic human exposure, and efficacy/safety separation should be quantified against an active comparator rather than inferred from weak *in-vitro* agonism.

### 6.3. Human Biomarkers That Anchor the Axis

Pioglitazone studies establish what target-informed human phenotyping can look like. Short-term treatment increased total and high-molecular-weight adiponectin alongside clamp-measured insulin sensitivity and adipose differentiation markers in insulin-resistant participants [55]. In drug-naive type 2 diabetes, pioglitazone increased plasma adiponectin, skeletal-muscle AMPK/ACC phosphorylation, insulin-stimulated glucose disposal, and expression of AdipoR1/AdipoR2 and oxidative genes [56]. Natural-product trials need not reproduce every measure, but they should prespecify a coherent subset.

A pragmatic proof-of-mechanism design would combine plasma and metabolite pharmacokinetics; total and high-molecular-weight adiponectin; fasting and postprandial free fatty acids; a validated insulin-sensitivity measure; edema and body-composition monitoring; and, where feasible, adipose biopsy for Ser273 phosphorylation, acetylation state, and a limited receptor-responsive transcript panel. The clinical product must be the same formulation and batch specification used in toxicology and mechanistic studies. The corresponding human benchmarks are summarized in Table 4.

## 7. Translational Failure Modes

The most common failure is a break in the evidence chain. A compound may dock to PPARγ but fail to bind experimentally; bind but not reach adipose tissue; activate a reporter but promote excessive adipogenesis; increase adiponectin *in vitro* but be rapidly conjugated in vivo; improve rodent glucose tolerance through an unrelated intestinal mechanism; or show a clinical glycemic signal in a product whose active chemistry differs from the material used in mechanistic studies. Each break can create an apparently coherent story from results that do not belong to the same pharmacological entity.

### 7.1. Assay Identity and Receptor Causality

Reporter assays should include full-agonist, partial-agonist, antagonist, and cytotoxicity controls, and their concentrations should be compared with direct binding and unbound cellular exposure. PPARγ dependence requires more than a correlation with receptor expression. Antagonist blockade, knockdown, receptor mutants, or rescue experiments should connect the candidate to the phenotype. Because many polyphenols affect AMPK, redox state, mitochondria, and membrane transport independently of PPARγ, glucose uptake that survives receptor disruption should be attributed to a parallel pathway rather than forced into a PPARγ narrative.

Cofactor recruitment and transcriptional profiling are essential for selective modulators. Two ligands with similar reporter efficacy can stabilize different receptor conformations and produce different gene sets. The desired profile is not universal: increased adipocyte lipid storage can be metabolically protective when it diverts lipid from the liver and muscle, whereas uncontrolled adipogenesis and fluid retention are liabilities. Consequently, adipogenesis, adipocyte size, lipolysis, lipid spillover, and tissue insulin sensitivity should be evaluated together.

Assays should also report whether the tested concentration perturbs membranes, redox-sensitive reporters, luciferase chemistry, or general transcription. Orthogonal confirmation is especially important for colored, fluorescent, aggregating, or redox-active natural products. A receptor-dependent phenotype should disappear or materially weaken when the receptor is disrupted; partial attenuation by an antagonist alone is not definitive because antagonists can have off-target effects and incomplete occupancy.

### 7.2. Exposure, Metabolism, and Tissue Targeting

Many natural PPARγ candidates are lipophilic, extensively metabolized, or present at low abundance. *In-vitro* experiments commonly use nominal micromolar concentrations without measuring unbound compound, precipitation, protein binding, or intracellular accumulation. Human development requires oral absorption, metabolite profiling, adipose and liver distribution, and a comparison between free tissue concentration and the concentration that produced receptor modulation. Active metabolites should be tested because conjugation can abolish, preserve, or redirect activity.

Nominal concentration is particularly misleading for lipophilic natural molecules. The pharmacologically relevant quantity is the free concentration at the receptor-containing tissue, which depends on solubility, protein binding, membrane partitioning, transport, and metabolism. Cell-culture media, serum content, plastic adsorption, and intracellular accumulation should therefore be documented when comparing potency with plasma or adipose exposure.

Local delivery can establish tissue mechanism while bypassing oral pharmacology, as illustrated by diosmin. It should be described as proof of local target engagement rather than evidence for an oral supplement. If formulation enhancement is required, the formulation itself becomes part of the pharmacological entity and needs separate distribution, safety, and interaction testing.

### 7.3. Product Reproducibility

For botanical products, species authentication is only the first step. Plant part, harvest conditions, extraction solvent, drug-to-extract ratio, excipients, stability, and quantitative markers must match across mechanistic, toxicological, and clinical studies. A chromatographic fingerprint should be accompanied by assays that are sensitive to the proposed active fraction. If the product is standardized to a marker unrelated to PPARγ activity, chemical consistency does not guarantee pharmacological consistency.

For a purified constituent, stereochemistry, polymorphism, oxidation, and degradation can change receptor behavior. For an extract, quantitative fingerprinting should cover the active fraction rather than only a convenient regulatory marker. Batch acceptance criteria should include a pharmacology-linked bioassay after analytical identity is established. These requirements are especially important in natural-product research, where the chemical definition and pharmacological mechanism must remain linked across mechanistic, toxicological, and translational studies.

### 7.4. Safety Must Be Mechanism-Matched

The adverse effects that motivate selective modulation should be prospectively measured rather than inferred from weak agonism. Relevant endpoints include body weight and fat distribution, food intake, plasma volume and edema, cardiac function in susceptible models, bone formation and resorption, liver injury, reproductive toxicity, and tumor-promoting signals where biologically plausible. A compound that does not cause lipid accumulation in one adipocyte line has not been shown to avoid fluid retention or bone loss.

Botanical mixtures add conventional toxicological concerns: reactive constituents, contaminants, CYP or transporter interactions, and additive hypoglycemia with established drugs. Because PPARγ is expressed in immune and stromal compartments, chronic modulation may alter inflammation, fibrosis, or tissue remodeling outside glucose metabolism. A selective transcriptional profile can therefore create new liabilities even while avoiding those of full agonists.

Ser273 blockade is not automatically beneficial in every tissue. Genetic work has revealed context-dependent hepatic effects despite improved insulin sensitivity, underscoring the need to evaluate liver lipid handling, inflammation, and fibrosis rather than extrapolating from adipose biomarkers alone [57]. Selective modulation should be presented as an experimentally testable benefit–risk hypothesis, not as a safety certificate.

## 8. A Seven-Stage Translational Roadmap

The proposed roadmap treats translation as a sequence of linked decisions. Each stage must use the same defined compound, extract, metabolite, or formulation, and every transition has a stop criterion. This design prevents an evidence chain from being assembled retrospectively from chemically different products, unrelated assays, or unattainable concentrations. The seven-stage sequence is shown in Figure 3, and the minimum decision package is detailed in Table 5.

## 9. Research Priorities

First, natural-product screens should move from single reporter readouts to multidimensional ligand profiling. Direct binding, cofactor recruitment, receptor phosphorylation, transcriptomics, and matched adipogenesis/glucose-uptake assays can identify receptor-state selectivity early. Structural work is especially valuable for compounds proposed to occupy the alternate Ω-loop-adjacent site.

Second, receptor-state assays should include the PPARγ2 and PPARγ1 numbering of the phosphorylation site, a direct measure of Ser273/Ser245 occupancy, K268/K293 acetylation, and a focused coregulator panel. Recent mechanistic reviews emphasize the therapeutic promise of phosphorylation blockade, but natural compounds should be tested against that standard rather than classified from docking alone [10].

Third, the adiponectin arm should be treated as a functional circuit. Studies should measure secretion of relevant adiponectin oligomers, AdipoR1/AdipoR2 dependence, AMPK and PPARα outputs, fatty-acid oxidation, and glucose transport in target tissues. This circuit can distinguish a receptor ligand that improves systemic communication from a compound that only changes adipocyte gene expression.

Fourth, pharmacokinetics must be integrated before extensive efficacy claims. Human-relevant intestinal metabolism, plasma protein binding, tissue distribution, and active conjugates should guide concentrations used in cell assays. For low-bioavailability compounds, formulation strategies should be justified by target location and safety rather than adopted solely to increase total plasma exposure.

Fifth, sex, age, circadian timing, and adipose depot should be incorporated earlier. PPARγ acetylation and adipose plasticity vary with metabolic state and time of day, and a compound may bias subcutaneous, visceral, or brown adipose programs differently [28,29]. These variables are not optional refinements when the proposed advantage is selective tissue remodeling.

Sixth, early clinical studies should avoid uninformative glucose-only designs. A short randomized proof-of-mechanism trial can measure exposure, adiponectin isoforms, fasting and postprandial glucose, insulin sensitivity, lipids, fluid status, and prespecified safety markers. The same standardized product must be used in the preceding pharmacology and toxicology package. This design is more demanding than a small uncontrolled supplement trial, but it directly tests the proposed advantage of selective modulation.

Seventh, negative results and exposure failures should be published. Demonstrating that a promising *in-vitro* ligand does not reach adipose tissue, that an extract changes composition across batches, or that a biomarker fails to move in humans is highly informative for the field. Transparent discontinuation criteria will reduce repeated investment in mechanisms that cannot be connected to a deliverable product.

## 10. Limitations of the Evidence Base and This Review

This is a critical narrative review and not a systematic review or meta-analysis. The heterogeneous literature spans purified proteins, reporter systems, multiple cell lineages, rodent models, locally delivered compounds, multi-constituent extracts, and small human studies, precluding quantitative pooling. The search and evidence hierarchy were designed to expose mechanistic discontinuities, but absence of a cited study should not be interpreted as proof of absence. Because this review used a targeted narrative rather than an exhaustive systematic search, candidate inclusion may be influenced by the availability of mechanistic studies and by citation-network visibility. The evidence ceilings assigned here are qualitative developmental descriptors rather than formal certainty-of-evidence grades and were not generated using a validated risk-of-bias instrument. They should therefore be interpreted as comparative indicators of how far a chemically defined candidate has progressed along the receptor-to-patient evidence chain. This choice reflects the scope and structure of the evidence base, which spans biochemical, structural, cellular, animal, and early clinical designs for which no single validated risk-of-bias instrument provides a comparable cross-design score. We therefore used a transparent mechanistic-continuity framework rather than combining non-equivalent domain ratings; this approach does not replace design-specific risk-of-bias assessment.

The natural-product field is also vulnerable to publication bias, batch-specific chemistry, incomplete reporting of negative assays, and over-reliance on nominal concentrations. Evidence grades in this review therefore denote the highest connected level for a defined material, not intrinsic efficacy or safety. Clinical recommendations cannot be made for the natural candidates on the basis of the evidence summarized here. Publication barriers for null or development-stopping results may further distort the evidence base, particularly when *in-vitro* activity occurs only at concentrations above attainable unbound in-vivo exposure, an extract loses activity across batches, a candidate fails to alter the proposed receptor-state biomarker in vivo, or an apparent effect disappears under receptor-dependence controls. Underreporting such findings can preferentially preserve positive but disconnected evidence chains and inflate the apparent maturity of the field.

## 11. Conclusions

The PPARγ–adiponectin axis remains a compelling route to insulin sensitization, but maximal receptor activation is neither necessary nor necessarily desirable. Natural products have contributed several mechanistically informative ligand classes. Amorfrutins and honokiol show that partial or selective activation can improve metabolic phenotypes with reduced adipogenic signals in preclinical systems; diosmin and betulinic acid broaden the concept to phosphorylation blockade and antagonism with preserved glucose uptake; valerenic and punicic acids show that adiponectin and GLUT4 benefits can coexist with some adipogenic activity. These distinctions are more useful than a binary agonist-versus-non-agonist label.

The field’s main limitation is not shortage of active molecules but shortage of connected evidence. Direct binding, receptor-state pharmacology, adiponectin/AdipoR function, clinically plausible exposure, product standardization, and mechanism-matched safety rarely appear in the same program. A natural candidate should advance only when those elements converge. Until then, the compounds discussed here are best regarded as drug-discovery scaffolds and mechanistic probes rather than substitutes for established insulin-sensitizing therapy.

Purified monomeric natural compounds can advance as medicinal-chemistry leads when chemical structure, stereochemistry, direct binding, receptor-state pharmacology, exposure, and safety are linked; their development endpoint is an optimized, chemically defined drug candidate. Plant extracts instead require botanical authentication, bioassay-guided fractionation, quantitative fingerprinting, batch-to-batch pharmacological equivalence, and mixture-specific toxicology; their endpoint is either an isolated active monomer or a standardized botanical product with reproducible composition and activity.

Preclinical findings should also be interpreted as model-specific: diet-induced obesity and db/db mice differ in leptin signaling, disease trajectory, and metabolic severity, so efficacy in one model should not be extrapolated to another without exposure-matched replication.

For translational development, the decisive contribution is not a longer list of phytochemicals but a falsifiable receptor-to-patient framework. A credible claim of selective natural modulation should identify the chemical entity, demonstrate direct and state-specific PPARγ engagement, connect it to adiponectin/AdipoR function, verify clinically plausible tissue exposure, and prospectively measure both the liabilities of full agonism and any new liabilities associated with the selected receptor state.

## Figures and Tables

**Figure 1:**
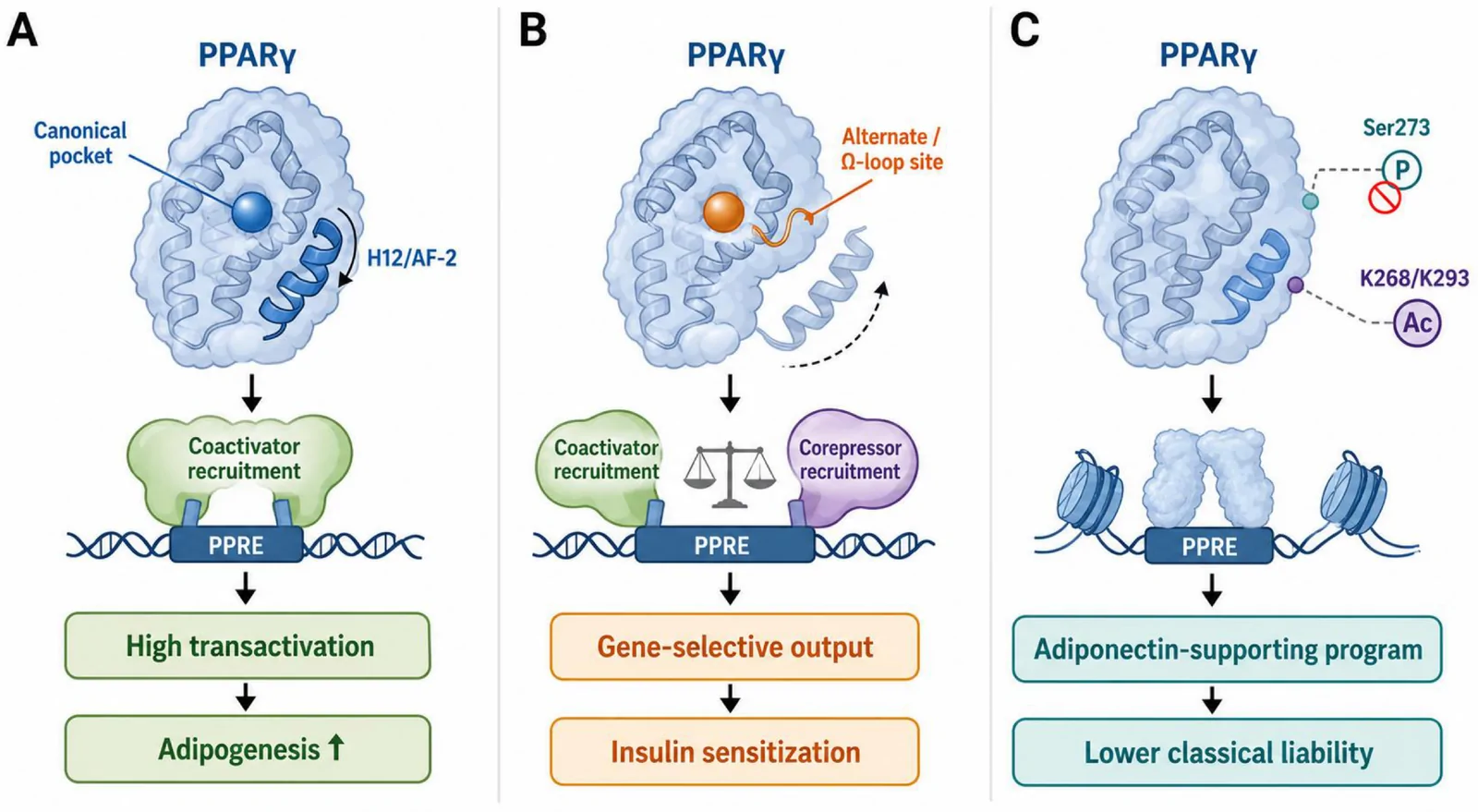
Receptor-state pharmacology of PPARγ. (**A**) Canonical full agonism stabilizes helix 12/AF-2, recruits coactivators, and produces high transactivation with adipogenic liability. (**B**) Partial or alternate-site modulation can generate a gene-selective cofactor balance. (**C**) modulation of Ser273 phosphorylation and the K268/K293 acetylation state can influence an insulin-sensitizing program without requiring maximal classical agonism. The panels represent pharmacological concepts, not effects established for every natural candidate.

**Figure 2:**
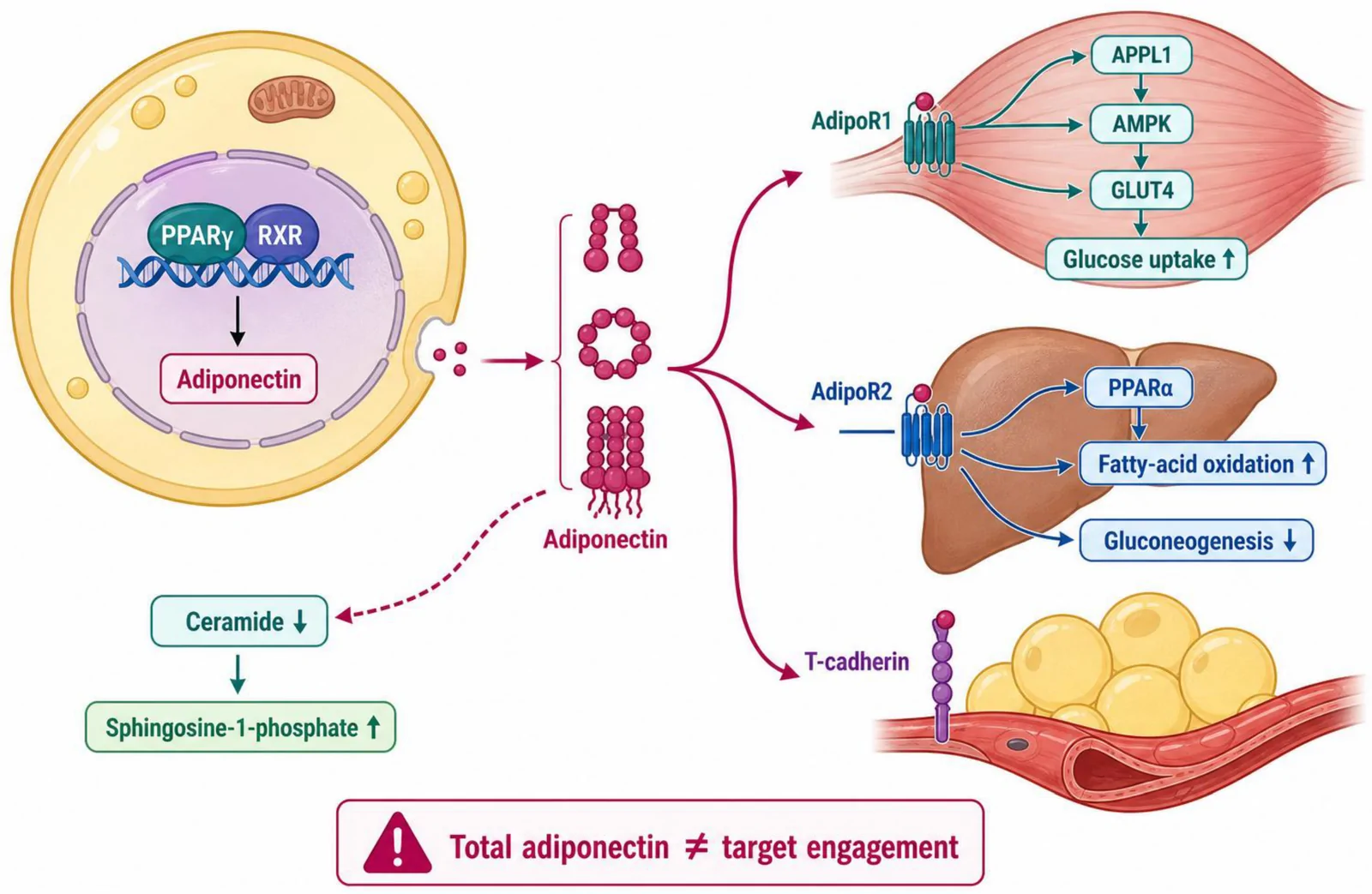
Adiponectin as a functional inter-organ output of PPARγ modulation. Adipocyte-derived adiponectin reaches AdipoR1 in skeletal muscle and AdipoR2 in liver, engaging APPL1–AMPK, GLUT4, PPARα, and ceramide/sphingosine signaling. T-cadherin contributes to tissue retention. Total circulating adiponectin is a useful pharmacodynamic clue but does not by itself establish PPARγ or AdipoR target engagement. AdipoR1, adiponectin receptor 1; AdipoR2, adiponectin receptor 2; APPL1, adaptor protein containing PH domain, PTB domain and leucine zipper motif 1; AMPK, AMP-activated protein kinase; GLUT4, glucose transporter type 4; PPARα, peroxisome proliferator-activated receptor alpha.

**Figure 3:**
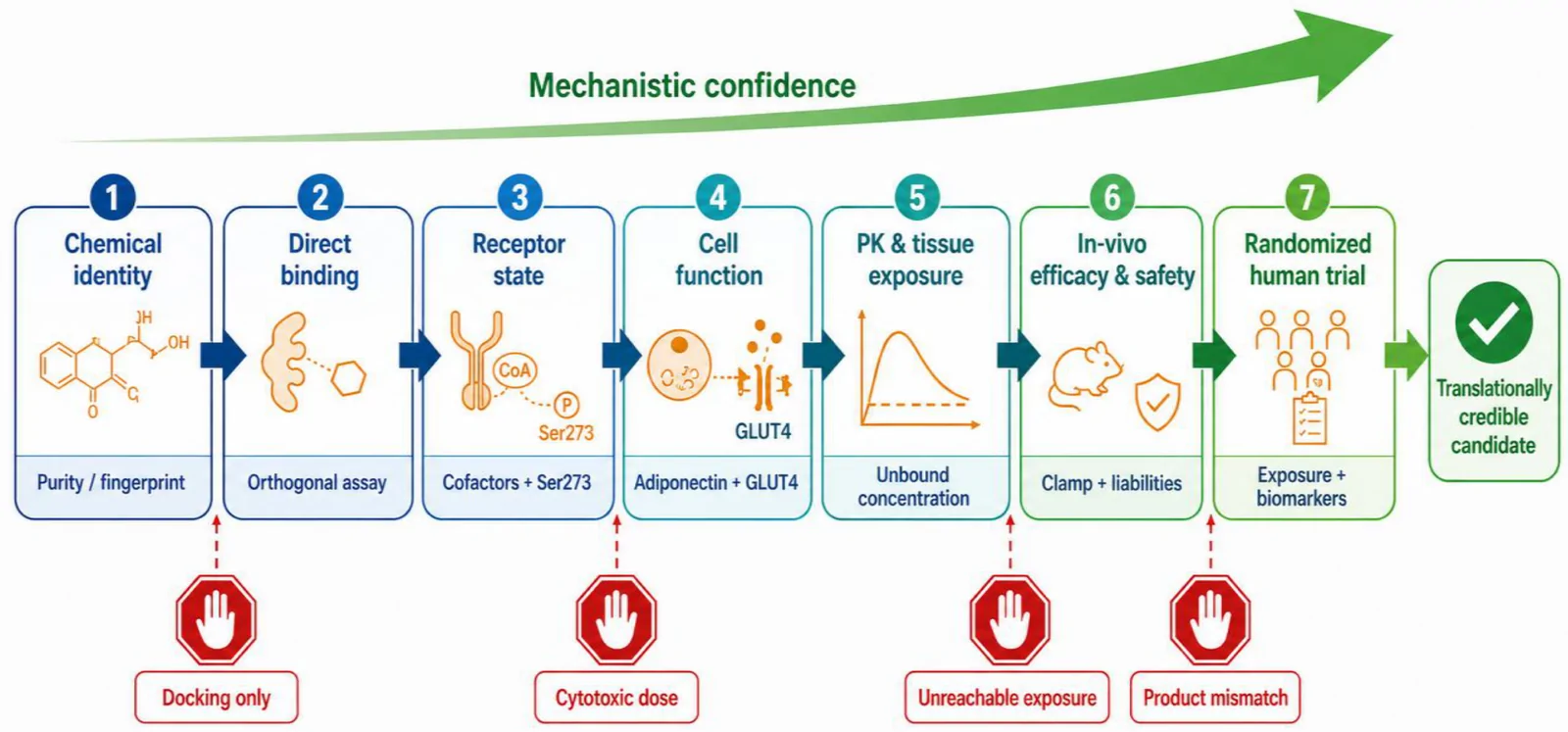
Translational roadmap for a natural PPARγ–adiponectin candidate. Mechanistic confidence increases only when chemical identity, orthogonal binding, receptor-state pharmacology, cell function, unbound tissue exposure, in-vivo efficacy/safety, and randomized human evidence are connected. Docking-only claims, cytotoxic concentrations, unreachable exposure, and product mismatch are explicit stop signals.

**Table 1:** Evidence levels used to assess natural modulators of the PPARγ–adiponectin axis.

Evidence Level	Preferred Measurements	What it Can Establish	Frequent Overinterpretation
Chemical identity	Purity, stereochemistry, quantitative marker panel, batch fingerprint	Tested entity and reproducibility of dose	Assigning an extract effect to an unquantified constituent; extract-level PPAR reporter and glucose-uptake screens [11,12] do not identify the responsible direct ligand
Direct engagement	Binding displacement, calorimetry, NMR, crystallography, orthogonal biophysics	Physical interaction and binding mode	Treating docking as target engagement
Receptor state	Transactivation, cofactor recruitment, Ser273 phosphorylation, K268/K293 acetylation, antagonist controls	Full, partial, selective, antagonistic, or non-agonist behavior	Equating increased PPARγ mRNA with agonism; valerenic acid illustrates that partial agonism can coexist with adipogenesis [13]
Cell function	Adiponectin secretion, GLUT4 transport, glucose uptake, lipid storage, cytotoxicity	Functional consequences in relevant cells	Using one reporter line as a metabolic phenotype
Organism and translation	Clamp studies, PK, tissue levels, edema, bone, heart, liver, randomized trials	Net metabolic benefit, safety, and plausible clinical exposure	Assuming high-dose rodent efficacy predicts human benefit

These complementary levels of evidence should be integrated rather than considered interchangeable.

**Table 2:** Experimental distinction between GPCR biased agonism and PPARγ receptor-state selectivity.

Dimension	GPCR Biased Agonism	PPARγ Receptor-State Selectivity
Mechanistic basis	Preferential stabilization of conformations that favor particular G-protein, β-arrestin, or other transducer pathways	Differential stabilization of nuclear-receptor conformations, coregulator recruitment, chromatin occupancy, and post-translational states
Reference comparison	Balanced reference agonist at matched receptor expression or occupancy	Full agonist and antagonist at matched receptor occupancy or unbound concentration
Primary indicators	Pathway-specific efficacy and potency, transducer recruitment, second messengers, internalization, and signaling kinetics	Direct binding, transactivation, coregulator recruitment, gene-selective transcription, Ser273/Ser245 phosphorylation, K268/K293 acetylation, adipogenesis, adiponectin, and glucose transport
Critical controls	Receptor reserve, signal amplification, assay timing, and kinetic bias	Receptor dependence, target engagement, exposure, assay interference, and off-target signaling

**Table 3:** Representative natural candidates selected on the basis of experimentally supported PPARγ engagement or receptor-state pharmacology, strongest functional evidence, and current translational ceiling.

Candidate/Source	Receptor-State Evidence	Best Functional Evidence	Current Ceiling/Decisive Next Step	Phosphorylation-Site Notation
Amorfrutins; Glycyrrhiza/Amorpha	Direct binding; selective/partial modulation	Primary human adipocytes and insulin-resistant mice [39,40]	Advanced preclinical; human PK and randomized proof	Not reported
Honokiol; Magnolia-derived neolignan	Direct binding; partial agonism; possible RXR contribution	Glucose uptake with reduced adipogenesis; diabetic-mouse efficacy [41]	Advanced preclinical; oral target engagement and chronic safety	Not reported
Valerenic acid; Valeriana marker	Partial agonism; proposed Ω-loop-adjacent pose	Adiponectin, GLUT4, glucose uptake, and adipogenesis [13]	Cellular; exposure-matched in-vivo benefit/liability balance	Not reported
Diosmin	Direct binding; weak transcription; Ser273 blockade	Local adipose delivery improved insulin sensitivity and browning in mice [44]	Mechanistic in vivo; oral adipose exposure and efficacy	Ser273 (PPARγ2)/Ser245 (PPARγ1)
Betulinic acid	PPARγ/PPARα antagonism with unusual binding	Glucose uptake, anti-adipogenesis, osteogenesis [18]	Cellular; systemic efficacy and receptor-dependent causality	Not reported
Punicic acid; pomegranate seed lipid	PPARγ-associated agonist activity	Adiponectin, GLUT4 expression/translocation, glucose uptake [42]	Cellular; unbound exposure, oxidation, and human outcomes	Not reported
Chelerythrine	Direct binding; crystal structure (PDB 4Y29); weak classical agonism; selective cofactor profile; Ser273 phosphorylation blockade	Improved insulin sensitivity in db/db mice [43]	Advanced mechanistic preclinical; PK, chronic safety, tissue exposure, and human proof	Ser273 (PPARγ2)/Ser245 (PPARγ1)
Swietenia constituents/Echinacea alkamides	Ligand activity or partial agonism	Adiponectin-related genes and glucose uptake [49,50]	Cellular; PK, metabolites, and in-vivo confirmation	Not reported
Botanical extracts	Usually reporter or expression-level evidence	Variable glucose uptake or rodent glycemia [11,12,45,46,47,48]	Discovery stage; isolate actives and standardize the tested material	Not reported

The table represents a mechanistic benchmark set rather than an exhaustive catalogue of natural products reported to influence PPARγ.

**Table 4:** Human and clinical benchmarks for translating receptor-state pharmacology.

Intervention	Design/Population	Key Readouts	Interpretive Value
Pioglitazone	Short controlled studies in insulin-resistant or type 2 diabetic adults	Clamp insulin sensitivity, HMW adiponectin, adipose or muscle biomarkers [55,56]	Shows that the PPARγ–adiponectin circuit is measurable in humans
INT131	24-week randomized placebo- and active-controlled trial; *n* = 367	HbA1c, edema, weight, hemodilution [53]	Clinical proof of dose-dependent efficacy/liability separation
Balaglitazone	26-week randomized phase III trial; *n* = 409	HbA1c and classical PPARγ safety endpoints [54]	Partial agonism can translate, but does not eliminate class liabilities
Phytate	Three-month randomized crossover trial in 39 people with type 2 diabetes	Adiponectin and HbA1c [52]	Axis-associated nutritional evidence; direct PPARγ engagement not established
Natural PPARγ ligands	No complete target-confirmed randomized program identified	Needed: PK, receptor-state biomarker, adiponectin isoforms, insulin sensitivity	Critical evidence gap and priority for future trials

HbA1c, glycated hemoglobin, HMW, high-molecular-weight; PK, pharmacokinetics, PPARγ, peroxisome proliferator-activated receptor gamma. Even the strongest human benchmarks in Table 4 do not fulfill the complete mechanistic ideal of combining high-molecular-weight adiponectin with a receptor-dependent tissue readout; total adiponectin alone remains an axis-associated biomarker rather than proof of target engagement.

**Table 5:** Minimum decision package for stage-gated development.

Stage	Minimum Experiments	Go Criterion	Stop or Redesign Signal
1. Chemical definition	Structure, purity, stereochemistry, stability, quantitative fingerprint	Reproducible material and dose	Unstable or unquantifiable active fraction
2. Target pharmacology	Orthogonal binding; reporter; cofactors; Ser273; K268/K293; receptor-dependence controls	Defined ligand/receptor-state class	Docking-only evidence or assay interference
3. Cellular metabolism	Adiponectin isoforms; AdipoR signaling; GLUT4; glucose uptake; adipogenesis; cytotoxicity	Sensitization exceeds adipogenic/cytotoxic liability	Activity only at cytotoxic or aggregating concentration
4. In-vivo translation	Oral PK, metabolites, adipose/liver levels, clamp or robust tolerance testing	Exposure-matched efficacy and target biomarker	Efficacy only at unreachable dose
5. Integrated safety	Weight, fluid, heart, bone, liver, interactions, sex and duration	Differentiated benefit–risk profile	Classical liabilities or new tissue-specific toxicity
6. Human mechanism	Standardized formulation, PK, adiponectin isoforms, insulin sensitivity, prespecified safety	Product-specific pharmacodynamic response	No target-relevant exposure or biomarker change
7. Human efficacy	Randomized, blinded, active/placebo-controlled trial with product verification	Clinically meaningful efficacy with acceptable tolerability	Product mismatch, weak design, or unbalanced benefit–risk

Progression should be based on the complete evidence chain rather than isolated positive assays.

## Data Availability

No new data were created or analyzed in this study. Data sharing is not applicable.

## Abbreviations

The following abbreviations are used in this manuscript.

ACC	Acetyl-CoA carboxylase
AF-2	Activation function 2
GLUT4	Glucose transporter type 4
LBD	Ligand-binding domain
PPAR	Peroxisome proliferator-activated receptor
RXR	Retinoid X receptor
T2DM	Type 2 diabetes mellitus
AdipoR1/2	Adiponectin receptor 1/2
AMPK	AMP-activated protein kinase
HMW	High-molecular-weight
PK	Pharmacokinetics
PPRE	Peroxisome proliferator response element
SPPARM	Selective PPARγ modulator
TZD	Thiazolidinedione
